# Genomic Diversity of Pigeon Pea (*Cajanus cajan* L. Millsp.) Endosymbionts in India and Selection of Potential Strains for Use as Agricultural Inoculants

**DOI:** 10.3389/fpls.2021.680981

**Published:** 2021-09-07

**Authors:** Beatriz Jorrin, Marta Maluk, Nagvanti Atoliya, Shiv Charan Kumar, Danteswari Chalasani, Andrzej Tkacz, Prachi Singh, Anirban Basu, Sarma VSRN Pullabhotla, Murugan Kumar, Santosh Ranjan Mohanty, Alison K. East, Vinoy K. Ramachandran, Euan K. James, Appa Rao Podile, Anil Kumar Saxena, DLN Rao, Philip S. Poole

**Affiliations:** ^1^Department of Plant Sciences, University of Oxford, Oxford, United Kingdom; ^2^The James Hutton Institute, Dundee, United Kingdom; ^3^ICAR-Indian Institute of Soil Science, Bhopal, India; ^4^ICAR-National Bureau of Agriculturally Important Microorganisms, Mau, India; ^5^Department of Plant Sciences, School of Life Science, University of Hyderabad, Hyderabad, India

**Keywords:** *Bradyrhizobium*, *Ensifer (Sinorhizobium)*, pigeon pea *(Cajanus cajan)*, *nod* cluster, nodulation outer proteins (Nop), comparative genomics, India

## Abstract

Pigeon pea (*Cajanus cajan* L. Millsp. ) is a legume crop resilient to climate change due to its tolerance to drought. It is grown by millions of resource-poor farmers in semiarid and tropical subregions of Asia and Africa and is a major contributor to their nutritional food security. Pigeon pea is the sixth most important legume in the world, with India contributing more than 70% of the total production and harbouring a wide variety of cultivars. Nevertheless, the low yield of pigeon pea grown under dry land conditions and its yield instability need to be improved. This may be done by enhancing crop nodulation and, hence, biological nitrogen fixation (BNF) by supplying effective symbiotic rhizobia through the application of “elite” inoculants. Therefore, the main aim in this study was the isolation and genomic analysis of effective rhizobial strains potentially adapted to drought conditions. Accordingly, pigeon pea endosymbionts were isolated from different soil types in Southern, Central, and Northern India. After functional characterisation of the isolated strains in terms of their ability to nodulate and promote the growth of pigeon pea, 19 were selected for full genome sequencing, along with eight commercial inoculant strains obtained from the ICRISAT culture collection. The phylogenomic analysis [Average nucleotide identity MUMmer (ANIm)] revealed that the pigeon pea endosymbionts were members of the genera *Bradyrhizobium* and *Ensifer*. Based on *nodC* phylogeny and *nod* cluster synteny, *Bradyrhizobium yuanmingense* was revealed as the most common endosymbiont, harbouring *nod* genes similar to those of *Bradyrhizobium cajani* and *Bradyrhizobium zhanjiangense*. This symbiont type (e.g., strain BRP05 from Madhya Pradesh) also outperformed all other strains tested on pigeon pea, with the notable exception of an *Ensifer alkalisoli* strain from North India (NBAIM29). The results provide the basis for the development of pigeon pea inoculants to increase the yield of this legume through the use of effective nitrogen-fixing rhizobia, tailored for the different agroclimatic regions of India.

## Introduction

Pigeon pea (*Cajanus cajan* L. Millsp.) is grown by millions of resource-poor farmers in semiarid and tropical subregions of Asia and Africa as a major contributor to their food security (Mula and Saxena, 2010; Varshney et al., 2010). The initial domestication of pigeon pea was started in central India over 3,500 years ago, from its wild progenitor *Cajanus cajanifolius* (Vavilov, 1951; Saxena et al., 2014). Pigeon pea is the sixth most important legume in the world, representing 5% of the total pulse production (4.92 M ha), with India contributing more than 70% of the total (3.6 M ha) and harbouring a wide variety of cultivars (218 making up 73% of the total) (Saxena, 2006). It was estimated by the Food and Agriculture Organisation (FAO) that the worldwide annual production of pigeon pea in 2019 was 5.6 Mt, of which ~59% was produced by India alone [FAO statistics (www.fao.org/faostat)].

Pigeon pea is a perennial shrub normally cultivated as an annual crop and, in India, can be used in rotation and intercrop systems with different cereal crops. Moreover, pigeon pea develops a deep root system, making it drought tolerant. These traits encourage cultivation in rain-fed drylands, although the poor growth conditions (e.g., aridity, nutrient-poor soils) mean that yields remain low. Effective symbiosis may improve nitrogen (N) content in this pulse legume and, hence, seed quality and quantity. However, legume-rhizobium symbioses are sensitive to drought, and, therefore, N fixation can be inefficient (Serraj et al., 1999; Mula and Saxena, 2010; Varshney et al., 2012). Selecting from among the diversity of pigeon pea cultivars sown in India may lead to improved symbiotic partners, as in the case for other legumes like soybean (Yang et al., 2010). To increase pigeon pea yields, it is important to select superior rhizobial strains that perform well under a wide variety of various stresses. Such bacteria can be developed into pigeon pea inoculants, tailored to perform well under different agroclimatic conditions.

However, until now, genomic diversity studies have only been performed in countries in the American and African continents, showing that the preferred endosymbionts are *Bradyrhizobium* spp. In Trinidad and Tobago, the main symbiont was *Bradyrhizobium elkanii* (Ramsubhag et al., 2002), whereas, in the Dominican Republic, *Bradyrhizobium yuanmingense* dominated. In the Ivory Coast, two different clades can nodulate pigeon pea, one associated with the *B. elkanii* group and a second one later assigned as the new species *B. ivorense* (Fossou et al., 2016, 2020). Additionally, another new species isolated from pigeon pea in the Dominican Republic has been defined as *Bradyrhizobium cajani* (Araújo et al., 2017), illustrating the great diversity present within pigeon pea endosymbionts across the world. *Ensifer* (syn. *Sinorhizobium*) has been reported as a symbiont only rarely, but strains were isolated using pigeon pea as a trap plant in soybean fields in Brazil (Coutinho et al., 1999; Stepkowski et al., 2003). Diverse pigeon pea rhizobia have been reported in Indian soils and have a long history of usage as inoculants; nevertheless, rigorous diversity studies have not been performed on these endosymbionts. In this study, we applied a mechanistic-holistic approach to study the diversity of pigeon pea native endosymbionts across India.

The aim in this study was to characterise the pigeon pea endosymbiont population isolated from a diversity of soil types in South (Alfisols), Central (Vertisols), and North India (Inceptisols). To achieve this, we isolated representative Indian pigeon pea rhizobia, sequenced representative strains, assessed their ability to nodulate pigeon pea and promote its growth, and analysed their genetic and genomic features. We uncovered the diversity of this population and the relationship between pigeon pea and members of the genera *Bradyrhizobium* and *Ensifer*. Comparisons of symbiotic-related features and the putative proteomes of these strains reveal the preferred pigeon pea endosymbionts in India.

## Materials and Methods

### Strain Isolation From Nodules

Pigeon pea nodules were collected from three different regions in India: South India representing Alfisols (Telangana/Andhra Pradesh, Hyderabad University, HU strains), Central India representing Vertisols (Madhya Pradesh, Bhopal Rhizobia Pigeon pea, BRP strains), and North India, representing Inceptisols (Uttar Pradesh/Haryana/Punjab, National Bureau of Agricultural Important Microorganisms, NBAIM strains) (Supplementary Table S1). Nodules were surface sterilised by washing with ethanol (70%) for 1 min, followed by 2% sodium hypochlorite for 5 min, and finally washing with sterile-distilled water. The nodules were homogenised in 0.9% NaCl and directly streaked on Yeast Mannitol media (YM), supplemented with Congo red (0.0025%) (CRYEMA) for visual screening (Vincent, 1970; Somasegaran and Hoben, 1994). Plates were incubated at 28°C for up to 3–5 days. Selected colonies were streaked onto fresh CRYEMA plates to obtain pure cultures.

Eight pigeon pea inoculant strains were obtained from the Microbial Germplasm collection of the International Crops Research Institute for the Semiarid Tropics (ICRISAT), Hyderabad, India, were also included as reference strains (Supplementary Table S1).

### Assessment of Bacterial Diversity by BOX-PCR

DNA extraction was achieved by alkaline lysis (0.05-M NaOH, 0.25% SDS) (Rivas et al., 2001). Isolated DNA was used as a template to generate BOX-PCR fingerprints, using the specific BOXA1R primer (CTACGGCAAGGCGACGCTGACG) (Versalovic et al., 1994). Amplification was carried out in a 25-μl PCR reaction containing 5–10 ng of isolated DNA and 1 U of OneTaq polymerase (NEB). BOX-PCR products were visualised on 2% agarose gels at 100 V until clear band separation. Gel images of the BOX-PCR fingerprint of each strain in the IU population were compared to find those that were the same and those that were different from each other.

### Nodulation Test

Seeds of *C. cajan* cv. Asha (ICPL 87119) were surface sterilised with sodium hypochlorite (3% active chlorine) and 0.1% (v/v) Tween 20 for 6 min and rinsed three times with sterile-distilled water. Surface-sterilised seeds were germinated on 0.5% distilled water agar in petri plates at 28°C in the dark. Germinated seedlings were transferred to sterile test tubes containing 30 ml of vermiculite: perlite mixture (1:1) and 30 ml of B&D nutrient solution. The tubes were transferred to a growth chamber at a temperature of 28°C, 16/8-h day/night light regime, a 70% moisture level, and 100 μmol m^−2^ s^−1^ irradiances. Each tube was inoculated with 1 ml of bacterial liquid culture (10^8^ CFU). Negative control tubes were left uninoculated. Five test tubes for each isolate were completely randomised in the growth chamber. Plants were harvested and scored for nodulation after 8 weeks of growth.

### Assessment of Plant Growth Promotion

A representative strain from each BOX-PCR pattern was used as an inoculant with pigeon pea to assess its plant growth-promoting potential, using a temperature of 28°C, 16/8-h day/night light regime, a 70% moisture level, and 100 μmol m^−2^ s^−1^ irradiances. The experiment was run as a completely randomised design with five replications. Sterile 1 L pots were filled with a 1:1 mixture of vermiculite: perlite and 400 ml B&D nutrient solution (Broughton and Dilworth, 1971). Seeds were surface sterilised and germinated as described above. Seedlings were transferred to 1-L pots and inoculated with 1 ml of bacterial liquid culture (10^8^ CFU). To prevent cross-contamination during watering, the pots were covered with plastic film with a hole for the shoot. Plants were fed weekly with a B&D nutrient solution and watered daily, or as required. The plants were harvested 8 weeks after inoculation, and shoot and root biomass obtained from five replicates was quantified after drying in an oven at 70°C for 5 days. The pigeon pea endosymbiont reference strains, IC3195, IC3342, IC4059, IC4060, and IC4062 were included as positive controls.

### Genome Sequencing, Annotation and Analysis

Culture samples were outsourced to Microbes NG, Birmingham, United Kingdom for Illumina sequencing (MiSeq v2, PE 2 × 250 bp). The closest available reference genome for each sample was identified with Kraken v2 (Wood and Salzberg, 2014), and reads were mapped to the reference genome using bwa-mem v0.7.17 (Li and Durbin, 2009) to assess the quality of the data. *De novo* assembly was performed with SPAdes v3.14.1 (Bankevich et al., 2012). Automated annotation was made using Prokka v1.12 (Seemann, 2014). Geneious R10 (v10.2.6) was used to investigate genome annotation. The rRNA copy number was estimated by calculating the relative coverage of 16S rRNA vs. that of *rpoB*, a single-copy gene. All genomes were uploaded to GenBank (BioProject PRJNA679722). BioSample IDs are given in Supplementary Table S2.

### Phylogenetic and Phylogenomic Analysis

*nodC* sequences from strains were extracted from annotated genomes or obtained from GenBank (Supplementary Table S3). Alignment was performed using MUSCLE software (Edgar, 2004). Distances were calculated according to the two-parameter model of Kimura (1980). Phylogenies of *nodC* were inferred using the neighbour-joining (NJ) method. All analyses were performed using MEGA X software (Kumar et al., 2018). All nodes with a bootstrap value lower than 70% were removed. The similarity of draught genome sequences of India-UK (IU) strains and ICRISAT (IC) strains (Table 1), together with genome sequences from closely related species considered as references, was analysed by calculating pairwise average nucleotide identity (ANI) values (Konstantinidis and Tiedje, 2005; Goris et al., 2007). ANI was performed using the Nucmer algorithm [Average nucleotide identity MUMmer (ANIm)] (Kurtz et al., 2004) as implemented in the JSpecies software v.1.2.1. Pairwise similarity percentage was transformed into a dissimilarity distance matrix and plotted as an NJ cladogram (Saitou and Nei, 1987) on MEGA X (Kumar et al., 2018). BioSample codes for each genome used can be found in Supplementary Table S3.

**Table 1:** Strains sequenced and used for genomic comparison.

**Species**	**Strain**	**Host**	**Location**
*Bradyrhizobium yuanmingense*	BRP05	*Cajanus cajan* cv Asha	Madhya Pradesh
*Ensifer* sp.	BRP08	*Cajanus cajan* cv Asha	Madhya Pradesh
*Bradyrhizobium yuanmingense*	BRP09	*Cajanus cajan* cv Asha	Madhya Pradesh
*Ensifer aridi*	BRP14	*Cajanus cajan* cv Asha	Madhya Pradesh
*Bradyrhizobium yuanmingense*	BRP19	*Cajanus cajan* cv Asha	Madhya Pradesh
*Bradyrhizobium yuanmingense*	BRP20	*Cajanus cajan* cv Asha	Madhya Pradesh
*Bradyrhizobium* sp.	BRP22	*Cajanus cajan* cv Asha	Madhya Pradesh
*Bradyrhizobium yuanmingense*	BRP23	*Cajanus cajan* cv Asha	Madhya Pradesh
*Bradyrhizobium brasilense*	BRP56	*Cajanus cajan* cv Asha	Madhya Pradesh
*Bradyrhizobium yuanmingense*	NBAIM01	*Cajanus cajan* cv Asha	Uttar Pradesh
*Bradyrhizobium yuanmingense*	NBAIM02	*Cajanus cajan* cv Asha	Punjab
*Bradyrhizobium yuanmingense*	NBAIM03	*Cajanus cajan* cv Asha	Punjab
*Bradyrhizobium yuanmingense*	NBAIM08	*Cajanus cajan* cv Asha	Uttar Pradesh
*Bradyrhizobium yuanmingense*	NBAIM14	*Cajanus cajan* cv Asha	Punjab
*Bradyrhizobium yuanmingense*	NBAIM16	*Cajanus cajan* cv Asha	Punjab
*Bradyrhizobium yuanmingense*	NBAIM18	*Cajanus cajan* cv Asha	Uttar Pradesh
*Bradyrhizobium yuanmingense*	NBAIM20	*Cajanus cajan* cv Asha	Uttar Pradesh
*Ensifer alkalisoli*	NBAIM29	*Cajanus cajan* cv Asha	Punjab
*Bradyrhizobium yuanmingense*	NBAIM32	*Cajanus cajan* cv Asha	Uttar Pradesh
*Bradyrhizobium yuanmingense*	IC4061	*Pongamia pinnata*	Uttar Pradesh
*Bradyrhizobium yuanmingense*	IC4060	*Pongamia pinnata*	Haryana
*Bradyrhizobium yuanmingense*	IC3069	*Indigofera glandulosa*	Telangana
*Bradyrhizobium yuanmingense*	IC4059	*Pongamia pinnata*	Tamil Nadu
*Bradyrhizobium yuanmingense*	IC3195	*Macroptilium atropurpureum*	Telangana
*Bradyrhizobium yuanmingense*	IC3123	*Aarachis hypogaea*	Maharashtra
*Ensifer* sp.	IC3342	*Macroptilium atropurpureum*	Telangana
*Ensifer* sp.	IC4062	unknown	Maharashtra

### Cluster Synteny

*nod* cluster regions were extracted from GenBank files using Geneious R10 (v10.2.6). Synteny analysis was performed in CloVR-Comparative (Angiuoli et al., 2011; Agrawal et al., 2017) and visualised with Sybyl in this platform (Riley et al., 2011). Sybyl defines an orthologue when a protein sequence has an identity >70%, a coverage cutoff of 80%, and an e-value >1^e−5^.

### Nodulation Outer Protein (Nop) Analysis

A local blast database was constructed with IU and IC draught proteomes. Well-characterised genes associated with Type 3 Secretion System (T3SS) machinery (*rhcQ, rhcU, ttsI, nolU*, and *nolV*) and its putative effectors (Nop: *nopA, nopB, nopC, nopD, nopE, nopF, nopJ, nopL, nopM, nopP, nopT, nopX, nopAA, nopAC*, and *nopAR*) were obtained from UniProt and NCBI databases (as shown in Supplementary Table S4), and blastp was performed. A blastp hit of at least 50% identity, 50% coverage, and e-value >1^e−5^ in protein sequence was considered an orthologue (as shown in Supplementary Table S4 for locus tags). Clustered heatmaps were generated using the pheatmap R package (Kolde, 2019).

### Genetic Features Analysis

CMG-Biotools were used to infer core genomes and pangenomes of IU and IC strains using for orthologue analysis protein files (Vesth et al., 2013). Protein files were uploaded to OrthoVenn2 running locally. Orthovenn2 uses a cutoff 1e^−5^ to define paralogues (within genomes) and orthologues (between genomes).

### Statistical Analyses

For PcoA plots construction, data were analysed in PRIMER 6 (PRIMER-E). Data were normalised and a similarity matrix was calculated using Euclidian distance. Strain samples that lacked a value in any tested variable were removed from the analysis. Permutational multivariate analysis of variance (PERMANOVA) was run in PRIMER 6 (PRIMER-E) using 9,999 unrestricted permutations of raw data. PERMANOVA produces pseudo-F values as a proxy for the difference between beta-diversity and alpha-diversity using a given factor. Statistical analyses were performed on PRISM 9 v9.0.2.

## Results

### Bacterial Diversity Revealed by BOX-PCR

**Figure 1:**
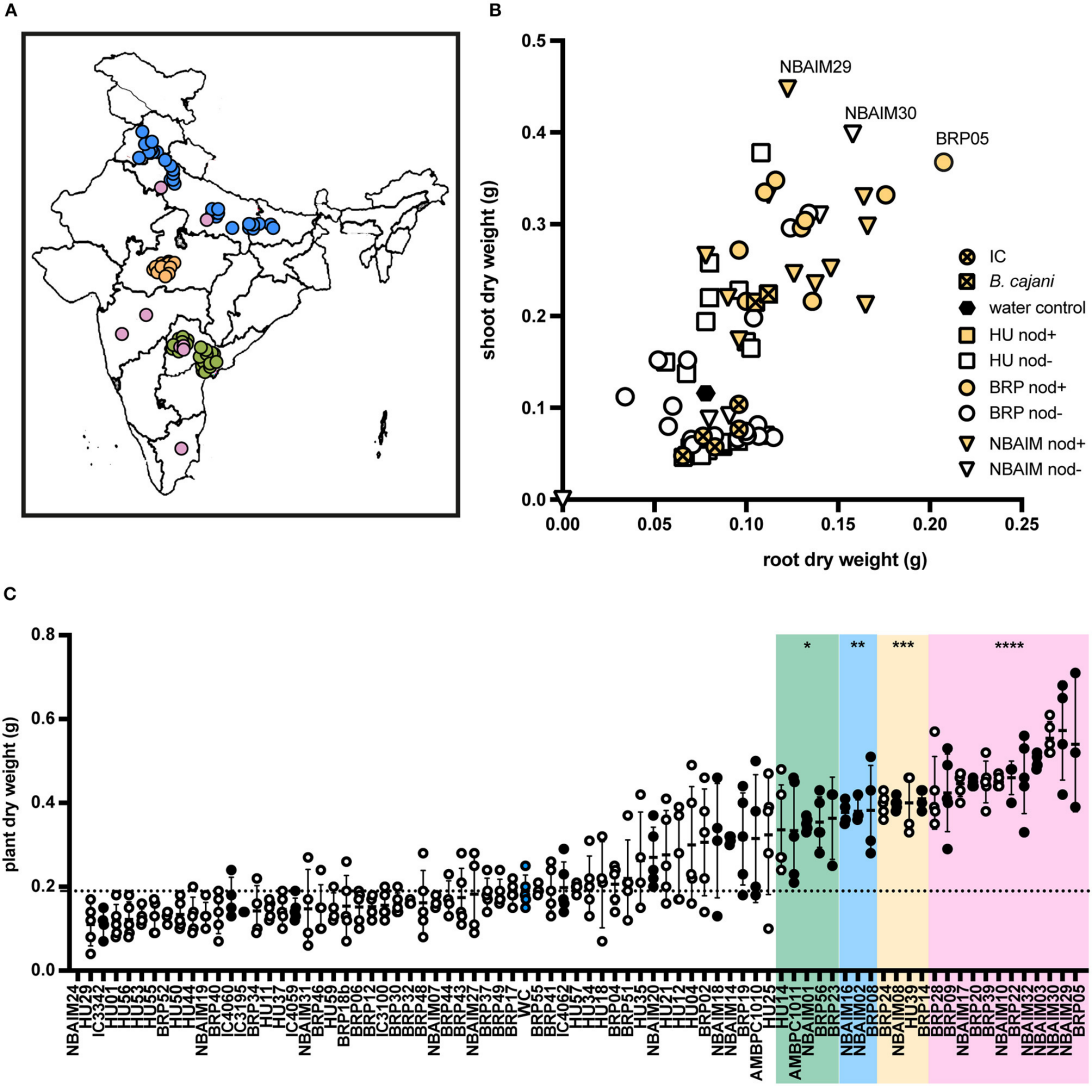
Isolation location and effect of bacterial inoculation on pigeon pea (*Cajanus cajan* L. Millsp.) of all India-UK (IU) strains and ICRISAT (IC) strains. **(A)** Map of India with isolation location for all IU and IC strains. In green, strains isolated by the University of Hyderabad (HU), yellow by ICAR-Indian Institute of Soil Science (BRP), blue by ICAR-National Bureau for Agriculturally Important Microorganisms (NBAIM), and pink ICRISAT pigeon pea reference strains. **(B)** Mean of dry weights of root (X-axis) and shoot (Y-axis) of *C. cajan* plants, following inoculation with bacterial strains. *B. cajani* reference strains AMBPC1010^T^ and AMBPC1011 are represented by crossed squares, IC strains by crossed circles, HU isolates by squares, BRP isolates as circles, and NBAIM isolates as triangles. White symbols represent non-nodulating strains (nod-), and yellow symbols show nodulating strains (nod+) under controlled conditions. **(C)** Mean plant dry weight for each strain. White circles represent nod- strains, black circles nod+, and blue circles water control. A horizontal dashed line represents the water control dry weigh average. One-way ANOVA analysis (*F* = 15.43, *R*^2^ = 0.8076, *p* < 0.0001) was performed followed by Sidak's *post-hoc* test. Asterisks represent a significance level vs. water control (WC) (*p < 0.05, **p < 0.01, ***p < 0.005, and ****p < 0.0001).

A total of 111 strains [termed collectively India-UK (IU) strains] were isolated from *C. cajan* root nodules in three different regions of India with different soils: 32 from South India (Alfisols; Telangana/Andhra Pradesh, HU strains), 47 from Central India (Vertisols; Madhya Pradesh, BRP strains), and 32 from North India (Inceptisols; Uttar Pradesh/Haryana/Punjab, NBAIM strains) (Supplementary Table S1, Figure 1A). BOX-PCR showed a total of 59 different profiles (a to bg) (Supplementary Table S5). A single representative strain from each region was selected from each BOX-PCR profile group, resulting in a total of 65: 20 HU strains, 27 BRP strains, and 18 NBAIM strains (Supplementary Table S5).

### Ability of Isolates to Nodulate and Influence Plant Growth

The 65 IU strains selected were used to investigate their effect on the growth of pigeon pea plants under controlled conditions (Supplementary Table S6). Some of these IC strains were included as positive controls since some of these are used as pigeon pea inoculants in India. In addition, *B. cajani* AMBPC1010^T^ and *B. cajani* AMBPC1011, isolated from *C. cajan* in the Dominican Republic (Araújo et al., 2017), were included as controls. Nineteen of the tested strains produced nodules on pigeon pea: 9 (BRP) and 10 (NBAIM) (Supplementary Table S6). None of the strains selected from the Telangana/Andhra Pradesh region (HU) formed nodules on *C. cajan* under these test conditions. Results varied from the increased dry weight of both root and shoot to a detrimental effect when inoculated with NBAIM24 where plants were dead (Figures 1B,C). It is clear that IC strains have a less beneficial effect on the growth of these plants than any of the IU-nodulating strains isolated in this study. Even though IC strains are used as pigeon pea inoculants across India, none were originally isolated from pigeon pea plants (Table 1) (Rupela et al., 1991). The strains that show the most significant increases in plant dry weight compared to uninoculated water controls are NBAIM29 (nod+) and BRP05 (nod+) (Figure 1C). It is important to mention that there are non-nodulating strains, which under these conditions promote the growth of pigeon pea by an unknown mechanism. The nod^−^ strain NBAIM30 has the best performance of a strain that does not form any nodules, with a positive effect on both shoot and root, outcompeting many nodulating strains (Figure 1C).

### Phylogenomic Diversity

**Figure 2:**
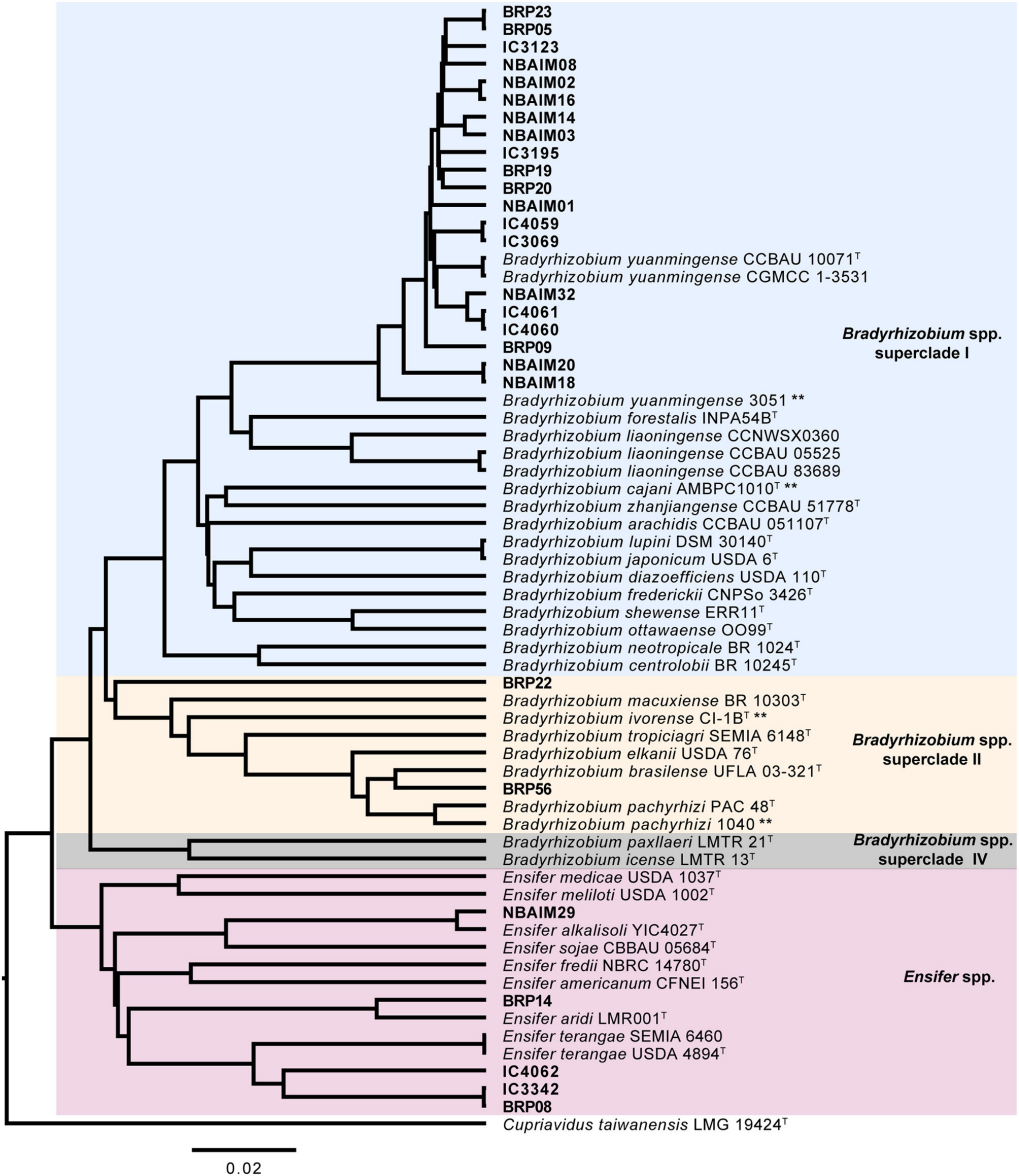
Average nucleotide identity MUMmer (ANIm) dendrogram. ANI-based UPMGA (unweighted pair group method with arithmetic mean) tree of IU, IC, and closely related reference strains. Bar, 2 nt substitutions per 100 nt. **Strains isolated from *C. cajan*.

We sequenced 27 genomes, 19 IU strains, which formed nodules under the test conditions, together with 8 IC strains (Table 1), for comparison with reference strains and to decipher the taxonomic diversity among them. Based on ANIm phylogeny, IU and IC strains were associated either with *Bradyrhizobium* (22 strains) or *Ensifer* (5 strains) (Figure 2). Twenty strains are related to *B. yuanmingense* CCBAU 10071^T^, showing ANIm similarity values 96.8–98.3%. They all show lower ANIm values (82.3–90.7%) with the next most similar type strains: *Bradyrhizobium forestalis* INPA54B^T^, *Bradyrhizobium liaoningense* CCBAU 83689^T^, *B. cajani* AMBPC1010^T^, and *Bradyrhizobium japonicum* USDA 6^T^ (Supplementary Table S7). We can consider that these twenty strains (BRP05, BRP09, BRP19, BRP20, BRP23, NBAIM01, NBAIM02, NBAIM03, NBAIM08, NBAIM14, NBAIM16, NBAIM18, NBAIM20, NBAIM32, IC3069, IC3965, IC3123, IC4059, IC4060, and IC4061) belong to *B. yuanmingense*; henceforth, they are defined as such in subsequent figures. Two strains cluster within *Bradyrhizobium* superclade II (Ormeño-Orrillo and Martínez-Romero, 2019). BRP56 has an ANIm of 96.5% with *Bradyrhizobium brasilense* UFLA03-321^T^, 95.5% with *B. elkanii* USDA 76^T^, and 95.4% with *Bradyrhizobium pachyrhizi* PAC 48^T^; henceforth, it is referred to as *B. brasilense* BRP56 in subsequent figures. Within the same superclade, BRP22 shows an ANIm similarity lower than 85.9% to all closely related type strains: *Bradyrhizobium macuxiense* BR 10303^T^, *B. ivorense* CI-1B^T^, *Bradyrhizobium tropiciagri* SEMIA 6148^T^, *B. elkanii* USDA 76^T^, *B. brasilense* UFLA 03-321^T^, and *B. pachyrhizi* PAC 48^T^. Strain BRP22 could represent a new species due to its ANIm similarity value lower than 96%, although new species descriptions based on a single strain are discouraged, given the requirement to demonstrate intraspecific diversity (De Lajudie et al., 2019). Therefore, we cannot, as yet, assign BRP22 to any given species, so it will subsequently be referred to as *Bradyrhizobium* sp. BRP22. For the five strains in the *Ensifer* group, NBAIM29 showed 98.9% similarity with *Ensifer alkalisoli* YIC4027^T^ (*E. alkalisoli* NBAIM29), BRP14 showed 95.8% similarity with *Ensifer aridi* LMR002^T^ (*E. aridi* BRP14), and BRP08, IC3342, and IC4062 showed just 91.2, 91.1, and 90.9% similarity, respectively, with the closest type strain, *Ensifer terangae* USDA 4894^T^, meaning that we cannot assign these latter three strains to any known species, i.e., *Ensifer* sp.BRP08, *Ensifer* sp.IC3342, and *Ensifer* sp.IC4062, respectively.

### Genome Characteristics

**Figure 3:**
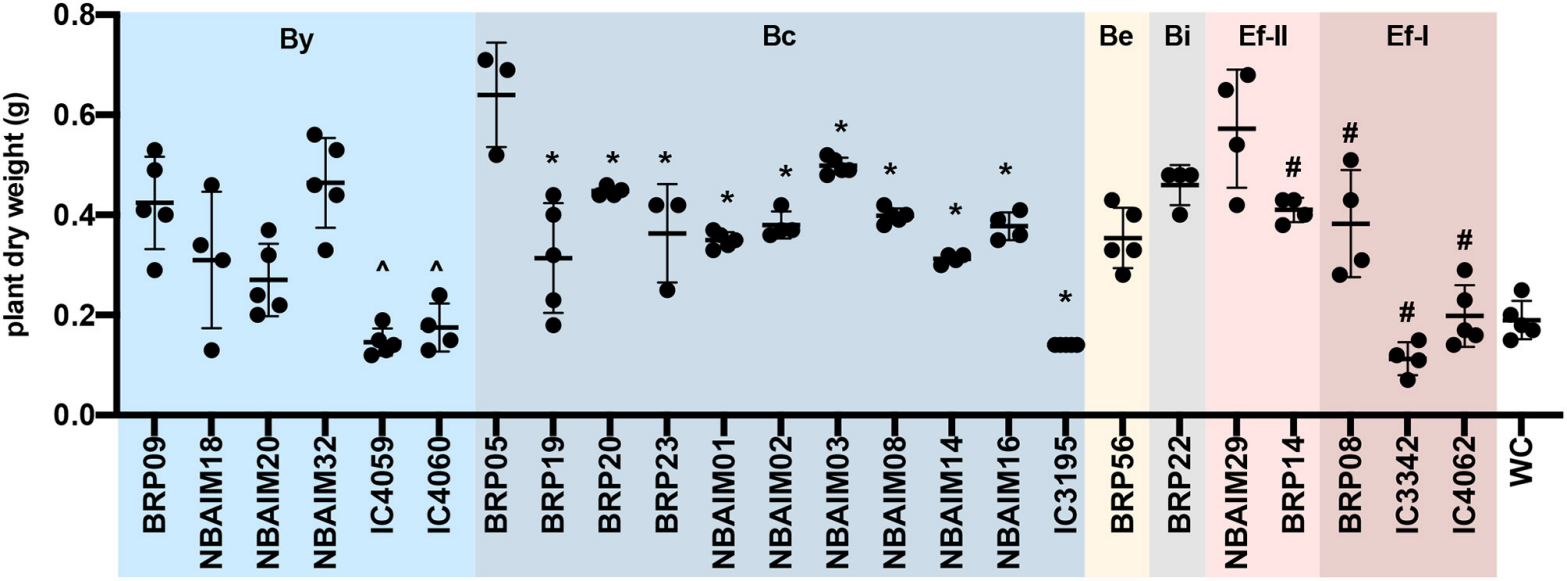
The dry weight of plants inoculated with IU and IC strains. Each dot represents a biological replicate. ∧Represents significant differences for each strain vs. BRP09 within the By group. *Represents significant differences for each strain vs. BRP05 within the Bc group. ^#^Represents significant differences for each strain vs. NBAIM29. Analysis was performed using one-way ANOVA (*F* = 17.51, *R*^2^ = 0.8318, *p* < 0.0001), with Sidak's *post-hoc* test. WC, water control.

The IU and IC *Bradyrhizobium* strains have a genome of 7.5–9.2 Mb, a GC-content >62.7%, absence of replication plasmid genes (*repABC*), and 20 out of 22 have a single estimated copy of 16S rRNA (Supplementary Table S2). Most *Bradyrhizobium* genomes range between 7 and 10 Mb with an average of 8.6 Mb (Ormeño-Orrillo and Martínez-Romero, 2019). However, *B. brasilense* BRP56 presents a remarkably larger genome among the IU and IC strains at 9.2 Mb (Supplementary Table S2). This strain is phylogenetically related to *B. elkanii*, which characteristically contains genomes larger than 9 Mb (Reeve et al., 2017). Since there is an inherent difficulty in resolving repetitive regions with short reads by assemblers (Waters et al., 2018), we estimate the rRNA copy number as the coverage ratio between 16S rRNA and the single-copy housekeeping gene *rpoB*. Most *Bradyrhizobium* IU and IC strains showed a single predicted copy, except for *B. yuanmingense* IC3069 and *B. yuanmingense* NBAIM32. Even though it is uncommon within the genus *Bradyrhizobium*, strains with closed genomes like *B. japonicum* USDA 6^T^ or *Bradyrhizobium* sp. BTAi1 have two copies of the rRNA cluster (Cytryn et al., 2008; Kaneko et al., 2011). There is a direct correlation between rRNA copy number and the time taken for a soil bacterium to respond to nutrient availability (Klappenbach et al., 2000), which could be translated into an adaptive advantage in a rhizosphere environment. In fact, *B. yuanmingense* NBAIM32 showed a significantly improved performance in plant growth experiments compared with other members of the By group (Figure 3, Supplementary Table S6). None of the IU and IC strains revealed the presence of plasmid-like replication genes (Supplementary Table S2). Although infrequent, plasmid presence was confirmed in *Bradyrhizobium* sp. BTAi1 and in *Bradyrhizobium* sp. DOA9 (Cytryn et al., 2008; Okazaki et al., 2015).

The IU and IC *Ensifer* strains showed a genome size of 6.5-7.4 Mb, 61-62 GC%, 3-6 estimated rRNA copies, and 2-3 plasmids (Supplementary Table S2). Most *Ensifer* spp. genomes have three copies of rRNA, as in *Ensifer fredii* NGR234 (Viprey et al., 2000), with the exception of *Ensifer* sp. IC4062, which shows six copies of rRNA. *Ensifer* IC strains have two copies of *repABC*, whereas IU strains have three, reflecting a different genomic organisation (Supplementary Table S2). These replicon numbers are in agreement with the work of Sugawara et al. (2013) with 48 different *Ensifer* spp., which showed 2–5 plasmids in Eckhart gels.

### Phylogeny-Based on *nodC*

**Figure 4:**
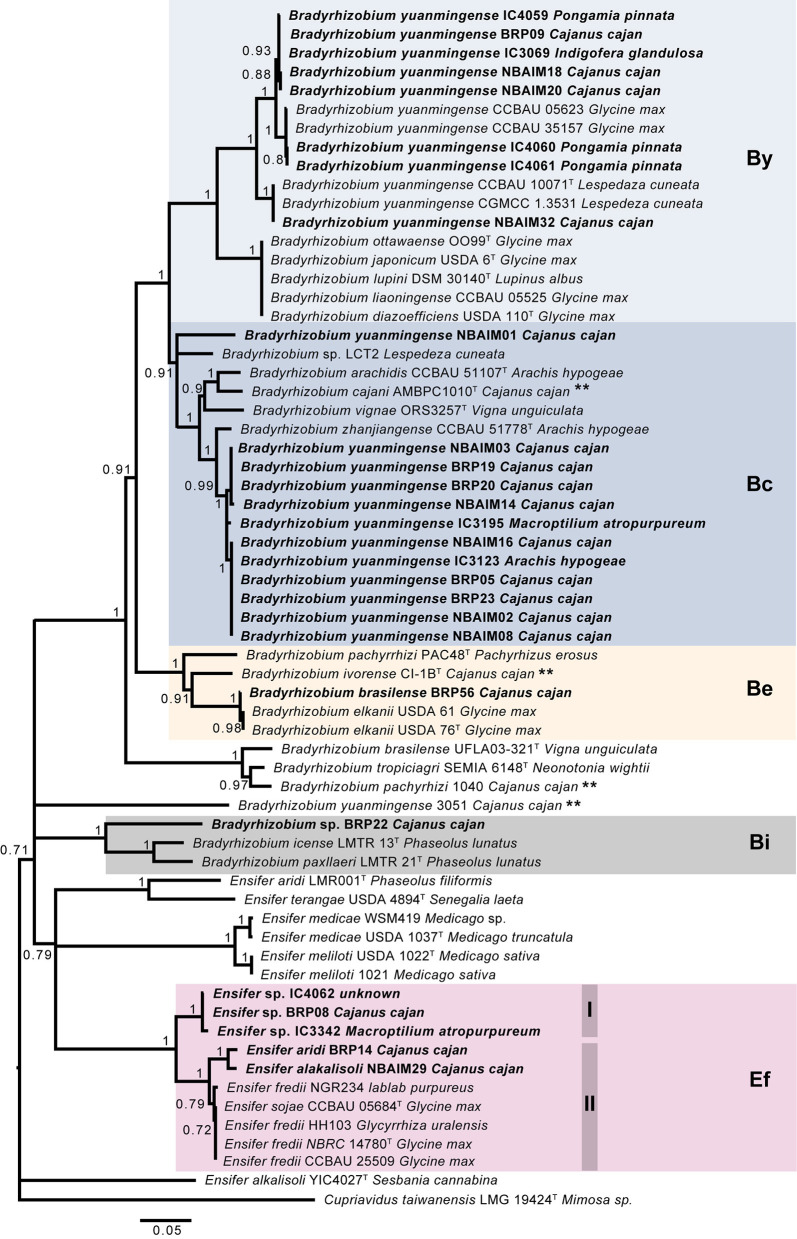
Tree-based on *nodC* phylogeny. Neighbour-joining (NJ) phylogenetic tree based on *nodC* sequence (1,458 nt) of IU and IC strains with closely related species. Each is shown together with the plant from which it was isolated. Bootstrap values (only values > 70%, expressed as a percentage of 1,000 replications) are shown at the branching points. Bar, 5 nt substitutions per 100 nt. **Strains isolated from *C. cajan*. By (*B. yuanmingense*), Bc (*B. cajani*), Be (*B. elkanii*), Bi (*B. icense*), and Ef (*E. fredii*).

The *nodC* phylogenetic tree (Figure 4) shows that the twenty-seven sequenced strains fall into five main groups: *B. yuanmingense* (By), 8 strains; *B. cajani* (Bc), 12 strains; *B. elkanii* (Be), 1 strain; *B. icense* (Bi), 1 strain; and *E. fredii* (Ef), 5 strains. The IU and IC strains assigned as *B. yuanmingense* belong to either the By or Bc group. In the By group, *B. yuanmingense* NBAIM32, NBAIM18, NBAIM20, BRP09, IC4060, IC4061, IC3069, and IC4059 show 95% *nodC* nucleotide identity with *B. yuanmingense* reference strains, which were isolated in China from *Glycine max* (soybean) or *Lespedeza cuneata*, among which is *B. yuanmingense* CCBAU 10071^T^. These IU and IC By strains share 89–90% *nodC* identity with the closed group formed by *B. diazoefficiens*-related strains (Supplementary Table S8). The second main group, Bc, comprises *B. yuanmingense* strains IC3195, IC3123, BRP19, BRP20, NBAIM14, NBAIM03, BRP05, BRP23, NBAIM02, NBAIM08, and NBAIM16, which showed more than 96.8% *nodC* nucleotide identity with *Bradyrhizobium zhanjiangense* CCBAU 51778^T^ and more than 92% with *B. cajani* AMBPC1010^T^ (Figure 4). *B. yuanmingense* NBAIM01 is more distant and shares 88.8–89.3% identity with other Bc, IU, and IC strains, and 87–87.7% with the aforementioned reference strains (Supplementary Table S8). The closest *nodC* sequence to that of *B. yuanmingense* NBAIM01 is from *Bradyrhizobium* sp. LCT2 (91.23%). It is within this Bc group that 44% of the sequenced strains clade together, showing that this is the most common *nodC* type found in Indian *C. cajan* endosymbionts. *B. brasilense* BRP56 (Be group) has a *nodC* very similar to that of *B. elkanii* strains (99.7% identity) and 91.3% with that of *B. ivorense* CI-1B^T^, a pigeon pea endosymbiont isolated in the Ivory Coast (Fossou et al., 2020). *Bradyrhizobium* sp. BRP22 is found in group Bi, with its *nodC* sequence, showing 83.2% and 81.9% similarity, respectively, to *B. icense* LMTR 13^T^ and *Bradyrhizobium paxllaeri* LMTR 21^T^ (Figure 4).

The IU and IC *Ensifer* strains have a *nodC* similar to that of *E. fredii* (Ef group), which clade in two subgroups, Ef-I (*Ensifer* sp. BRP08, IC3342, and IC4062) and Ef-II (*E. aridi* BRP14 and *E. alkalisoli* NBAIM29). Within Ef-I, the *nodC* similarity is 99.3–99.9% and <94% with *nodC* from Ef-II. The Ef-II IU strains share 98.4% *nodC* identity, and *circa*, 96%, with *E. fredii* and *E. sojae* reference strains in the same Ef-II group (Figure 4).

### *Nod* Cluster Synteny

*Nod* gene cluster synteny analysis was performed for strains in each *nodC* group: By, Bc, Be, Bi, and Ef (Figure 4). All IU and IC strains have *nodABCIJ* as a core cluster, which is present in all symbiotic nod factor (NF)-dependent rhizobia.

#### Bradyrhizobium

**Figure 5:**
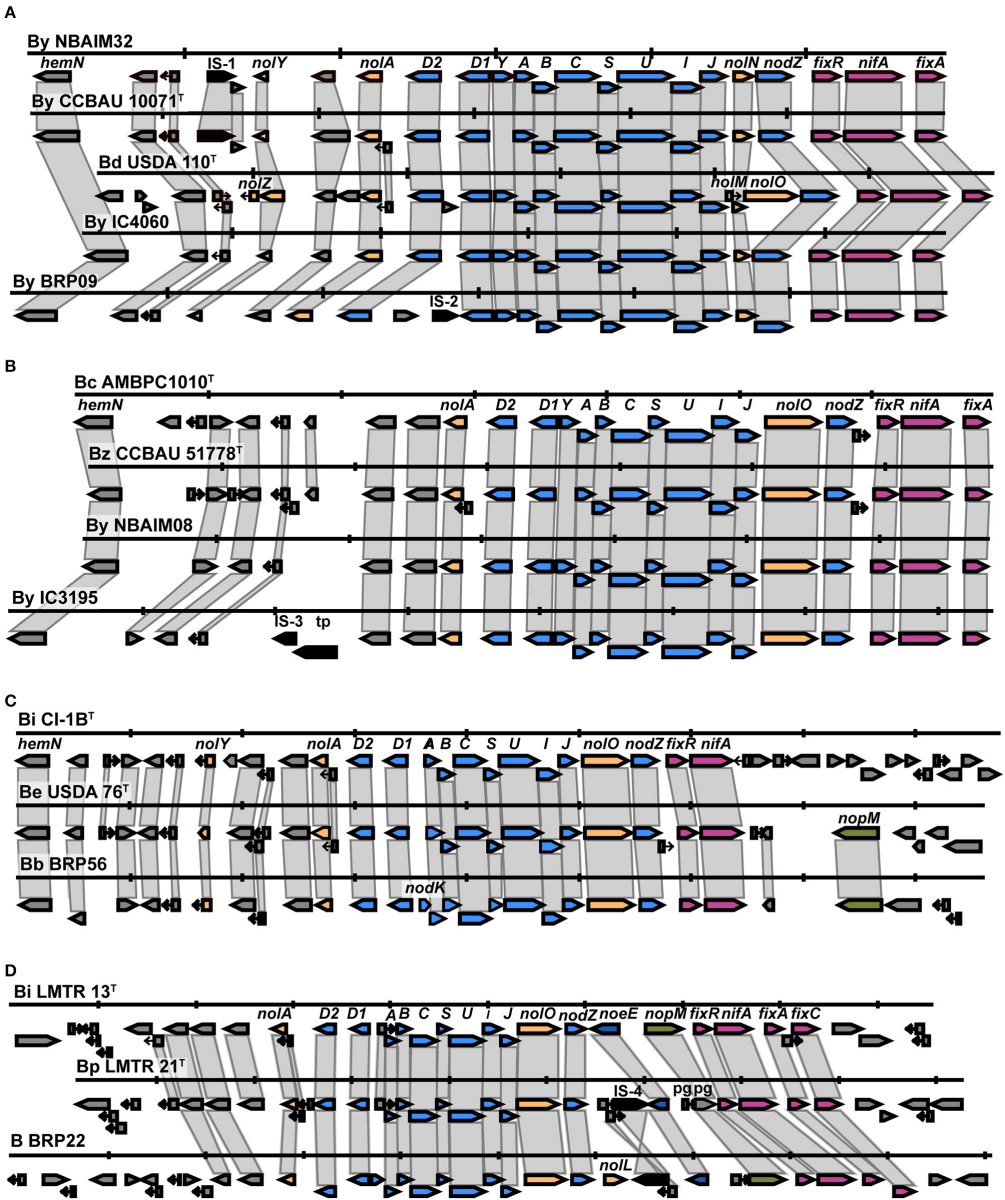
The *nod* cluster synteny of IU and IC strains in the By, Bc, Be, and Bi groups. **(A)** By group, **(B)** Bc group, **(C)** Be group, and **(D)** Bi group. Each row represents a single strain and shows the *nod* cluster organisation and its genomic context. The colour of the arrow reflects the genes: blue for *nod*, yellow for *nol*, pink for *fix-nif* , green for *nop*, dark blue for *noe*, and black for transposase/insertion-related genes. Vertical markers indicate 5 Kb in each genome. B (*Bradyrhizobium* sp.), By (*B. yuanmingense*), Bd (*B. diazoefficiens*), Bc (*B. cajani*), Bz (*B. zhanjiangense*), Bi CI-1B^T^ (*B. ivorense* CI-1B^T^), Be (*B. elkanii*), Bb (*B. brasilense*), Bi LMTR13^T^ (*B. icense* LMT13^T^) and Bp (*B. paxllaeri*).

All newly sequenced strains in the By group show the presence of the same nodulation-related genes, *nolY*-[]-*nolA*-[]-*D2*-[]-*D1YABCSUIJ*-*nolN*-*nodZ* (Supplementary Figure S1). Representative strains *B. yuanmingense* BRP09, *B. yuanmingense* IC4060, and *B. yuanmingense* NBAIM32 were selected to further investigate their synteny with *B. yuanmingense* CCBAU 10071^T^ and *B. diazoefficiens* USDA 110^T^ (Figure 5A). *B. diazoefficiens* USDA 110^T^ has three extra nodulation-related genes, *nolZ, nolM*, and *nolO*, not present in any of the newly sequenced strains. The By *nod* cluster is highly conserved, albeit with evidence of different insertion events. We can conclude that the By strains *B. yuanmingense* NBAIM18, NBAIM20, NBAIM32, BRP09, IC3069, IC4059, IC4060, and IC4061 have the same *nod* cluster as *B. yuanmingense* CCBAU 10071^T^. A highly conserved *nod* cluster, *nolA*-[]-*nodD2D1YABCSUIJ-nolO-nodZ*, is present in the newly sequenced strains belonging to the Bc group (Supplementary Figure S2). *B. yuanmingense* NBAIM08 and IC3195 were selected as representative and aligned with *B. cajani* AMBPC1010^T^ and *B. zhanjiangense* CCBAU 51778^T^ (Figure 5B). Notwithstanding transposase-related genes in *B. yuanmingense* IC3195 (which are not present in either of the type strains), we can conclude that the *nod* cluster and its genomic context are the same as that of *B. cajani* AMBPC1010^T^ and *B. zhanjiangense* CCBAU 51778^T^. The *nodC* phylogeny shows that *B. brasilense* BRP56 belongs to the Be group, together with *B. ivorense* CI-1B^T^ and *B. elkanii* USDA76^T^ (Figure 4). The observed *nod* cluster is *nolY*-[]-*nolA*-[]-*nodD2D1*-[]-*ABCSUIJ*-*nolO*-*nodZ* (Figure 5C). *B. brasilense* BRP56 has a *nodK* gene among *nodD1* and *nodA*, which is not annotated in CI-1B^T^, and neither is it in *B. elkanii* USDA 76^T^. Both reference strains have an open reading frame (ORF) in this region, which, in USDA 76^T^, has an amino acid (aa) identity of 97.7% to *nodK* of *B. brasilense* BRP56, whereas, in CI-1B^T^, it is just 59.4%. Furthermore, both *B. brasilense* BRP56 and *B. elkanii* USDA 76^T^ have *nopM* downstream of *nifA* (Figure 5C). Overall, we suggest that *B. brasilense* BRP56 has a typical *B. elkanii*-type *nod* cluster. *Bradyrhizobium* sp. BRP22, together with *B. icense* LMTR 13^T^ and *B. paxllaeri* LMTR 21^T^, belongs to the Bi *nodC* group (Figure 4). Analysis of their *nod* cluster synteny shows that all strains have *nolA*-[]-*nodD2D1*-[]-*ABCSUIJ-nolO-nodZ*-[]-*noeE* (Figure 5D). In addition, *Bradyrhizobium* sp. BRP22 and *B. icense* LMTR 13^T^ have *nopM* downstream of *noeE*, whereas *B. paxllaeri* LMTR 21^T^ shows a pseudogene (pg) with 50–54% aa identity to the N-terminal part of *nopM*. We conclude that *Bradyrhizobium* sp. BRP22 has a *B. icense*-type *nod* cluster.

In selected representative strains from each *Bradyrhizobium nodC* group (Figures 4, 5), this region of DNA was aligned (Supplementary Figure S3). As all strains have *nolA*-[]-*nodD2*-[]-*nodD1*-[]-*nodABCSUIJ*-[]-*nodZ*, this could be considered the minimum *nod* cluster necessary to nodulate pigeon pea. Other nodulation-related genes (*nolY, nodK, nodY, nolO, nolN, nolL*, or *noeE*) may be associated with host specificity and, therefore, play a part in the symbiotic performance.

#### Ensifer

**Figure 6:**
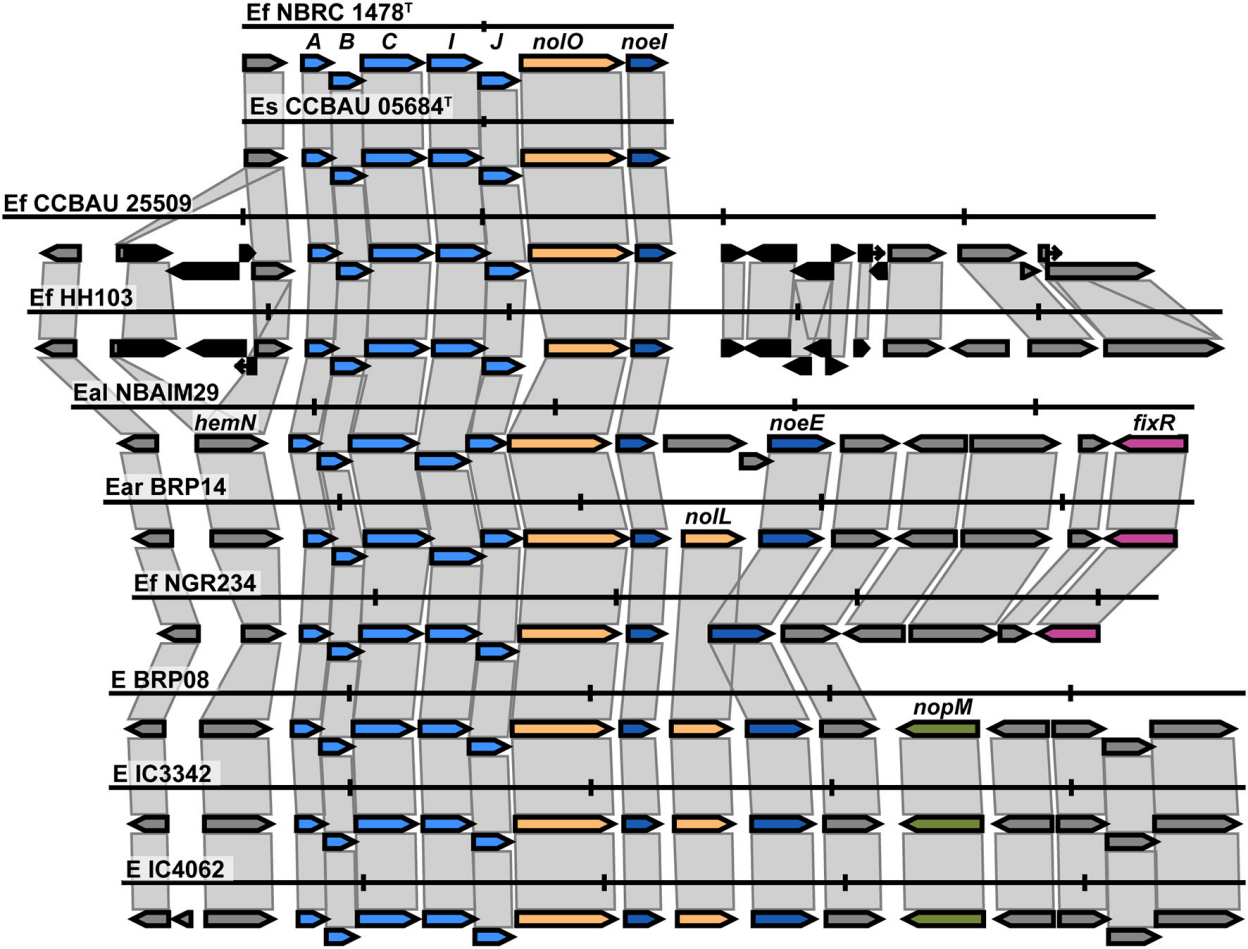
The *nod* cluster synteny of IU and IC strains in the *Ensifer* group together with type strains. Each row represents a single strain and shows the *nod* cluster organisation and its genomic context. The colour of the arrow reflects the genes: blue for the *nod*, yellow for *nol*, pink for *fix-nif* , and black for transposase/insertion-related genes. Vertical markers indicate 5 Kb in each genome. E (*Ensifer* sp.), Ef (*E. fredii*), Es (*E. sojae*), Eal (*E. alkalisoli*) and Ear (*E. aridi*).

The *nod* cluster regions for the five newly sequenced *Ensifer* strains were aligned and, together with reference strains in the same *nodC* phylogenetic clade, reveal a high degree of synteny within the group (Figure 6). The observed canonical *nod* cluster is *nodABCIJ-nolO-noeI*-[]-*noeE*. There are two genomic contexts for each *nod*C group (Ef-I and Ef-II), which suggests that they could have a different origin (Figure 6). Despite this, the IU and IC strains show great conservation of the *nod* cluster, except for the absence of *nolL* from *E. alkalisoli* NBAIM29. Instead, it has *cysNC* (adenylyl-sulphate kinase, A0A4S5J185). The lack of *nolL* could give *E. alkalisoli* NBAIM29 an advantage in plant recognition, which would explain its enhanced growth promotion phenotype observed *in planta* (Figure 3), although, as it is based only on a single strain, this is highly speculative.

### Presence of Gene-Encoding Nops

There are multiple pieces of evidence supporting a T3SS and Nops and their key roles in the establishment of symbiosis and host specificity in certain rhizobia-legume interactions (Pueppke and Broughton, 1999; López-Baena et al., 2016). We have confirmed the presence of T3SS machinery by finding orthologues (>50% aa identity and coverage) for *rhcQ, rhcU, ttsI, nolV*, and *nolU* from the well-characterised T3SS of *B. vignae* ORS3257 (Teulet et al., 2019) and *E. fredii* NGR234 (Freiberg et al., 1997) in all the newly sequenced strains presented in this study (Supplementary Table S4). To determine the putative range of T3SS effectors, we have based analysis on the Nops and used Nop sequences from well-characterised *Ensifer* and *Bradyrhizobium* spp. to find homologues in the IU and IC genomes (as shown in Supplementary Table S4).

**Figure 7:**
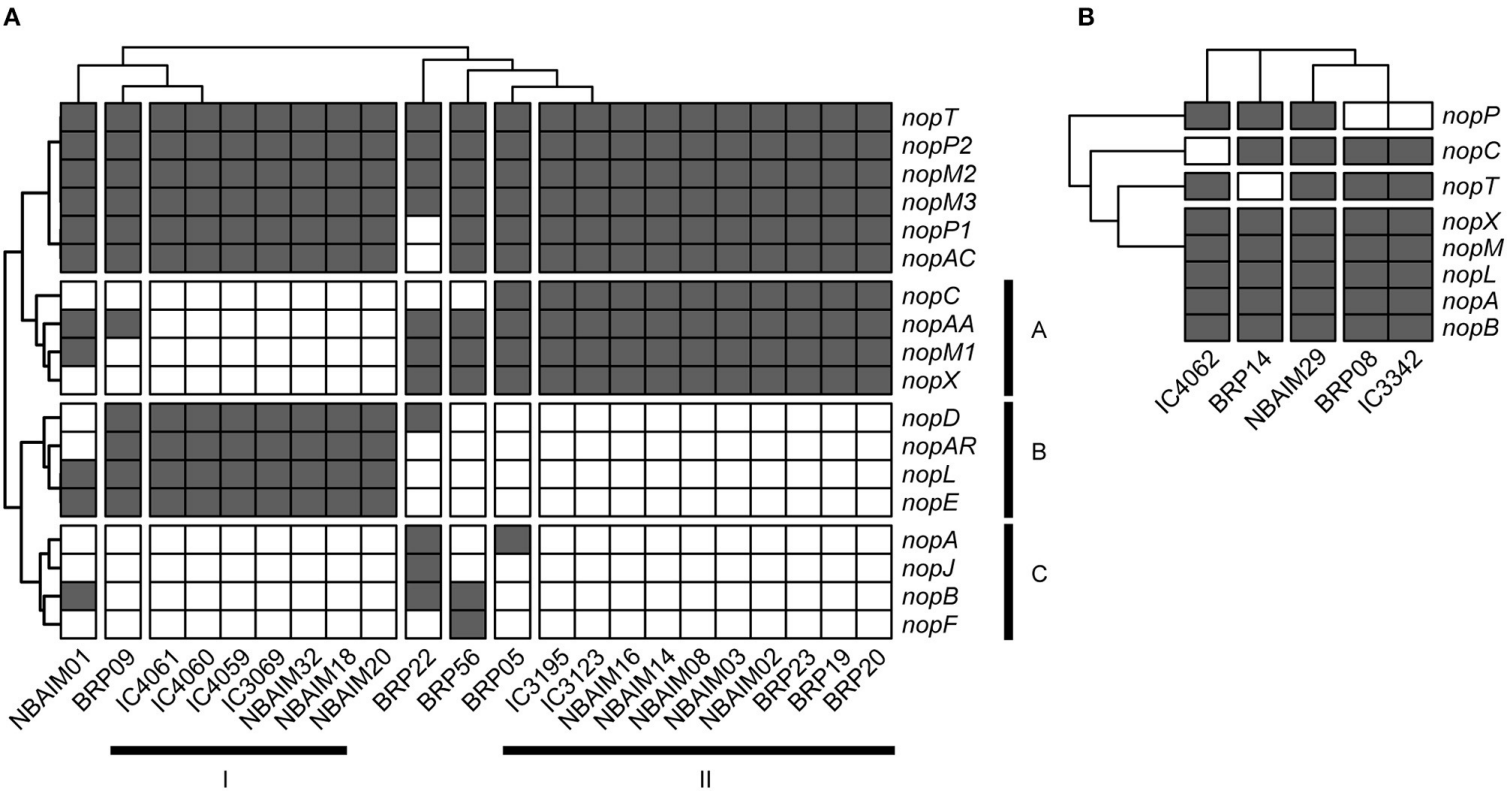
The presence and absence of *nop* genes. **(A)** *Bradyrhizobium* IU and IC strains are shown as a heat map. **(B)** *Ensifer* IU and IC strains are shown as a heat map.

Within the *Bradyrhizobium* strains, all IU and IC strains have orthologues for *nopT, nopP2, nopM2*, and *nopM3*. These T3SS effectors could be needed for establishing symbiosis between *Bradyrhizobium* spp. and pigeon pea (Figure 7A). The groups formed based on the presence and absence of Nop orthologues in *Bradyrhizobium* spp. (Figure 7A) are highly correlated with those observed in the *nodC* phylogeny and *nod* cluster synteny (Figures 4, 5). Cluster I (By) and cluster II (Bc) are distinguished from each other by the presence or absence of two groups of *nop* genes: group A (*nopC, nopAA, nopM1*, and *nopX*), group B (*nopD, nopAR, nopL*, and *nopE*), where cluster I (By) has group A genes but not those of group B and cluster II (Bc), vice versa (Figure 7A). There are a few orphan strains that present a different presence/absence pattern, e.g., *B. yuanmingense* BRP09 has all the Nop genes present in the other By strains (cluster I), plus *nopAA* (Figure 7A). However, we have not observed differences in plant dry weight between *B. yuanmingense* BRP09 and other IU strains of the By group (Figure 3), suggesting that the presence of *nopAA* is uncorrelated with plant performance. *Bradyrhizobium yuanmingense* NBAIM01 belongs to the Bc *nod* group (cluster II), although it shows a very different set of Nop homologues. Its lack of *nopX* and *nopC* (*nolJ*) could be counterbalanced by the presence of *nopB*, nopL, and/or *nopE* effectors since the plant performance of *B. yuanmingense* NBAIM01 is similar to that of other Bc members (Figure 3). Finally, *B. yuanmingense* BRP05 displays the common Nop genes for Bc strains (group II), plus *nopA* (Figure 7A). This strain promotes plant growth significantly more than any other Bc member or, indeed, any other *Bradyrhizobium* strains tested (Figure 3), which could be a result in part of the presence of this T3SS effector, although, without further strains showing similar characteristics, it is impossible to draw firm conclusions at this stage.

In comparison to *Bradyrhizobium*, a total of only seven *nop* genes are present in *Ensifer* (Figure 7B). All *Ensifer* IU and IC strains show orthologues for *nopA, nopB, nopL, nopM*, and *nopX*. Nevertheless, only *E. alkalisoli* NBAIM29 has *nopC, nopP*, and *nopT*, and, together with the lack of *nolL*, could equip it for improved performance on pigeon pea (Figure 3). However, further strains showing the same characteristics are required to test this speculation.

### Genetic Features

**Figure 8:**
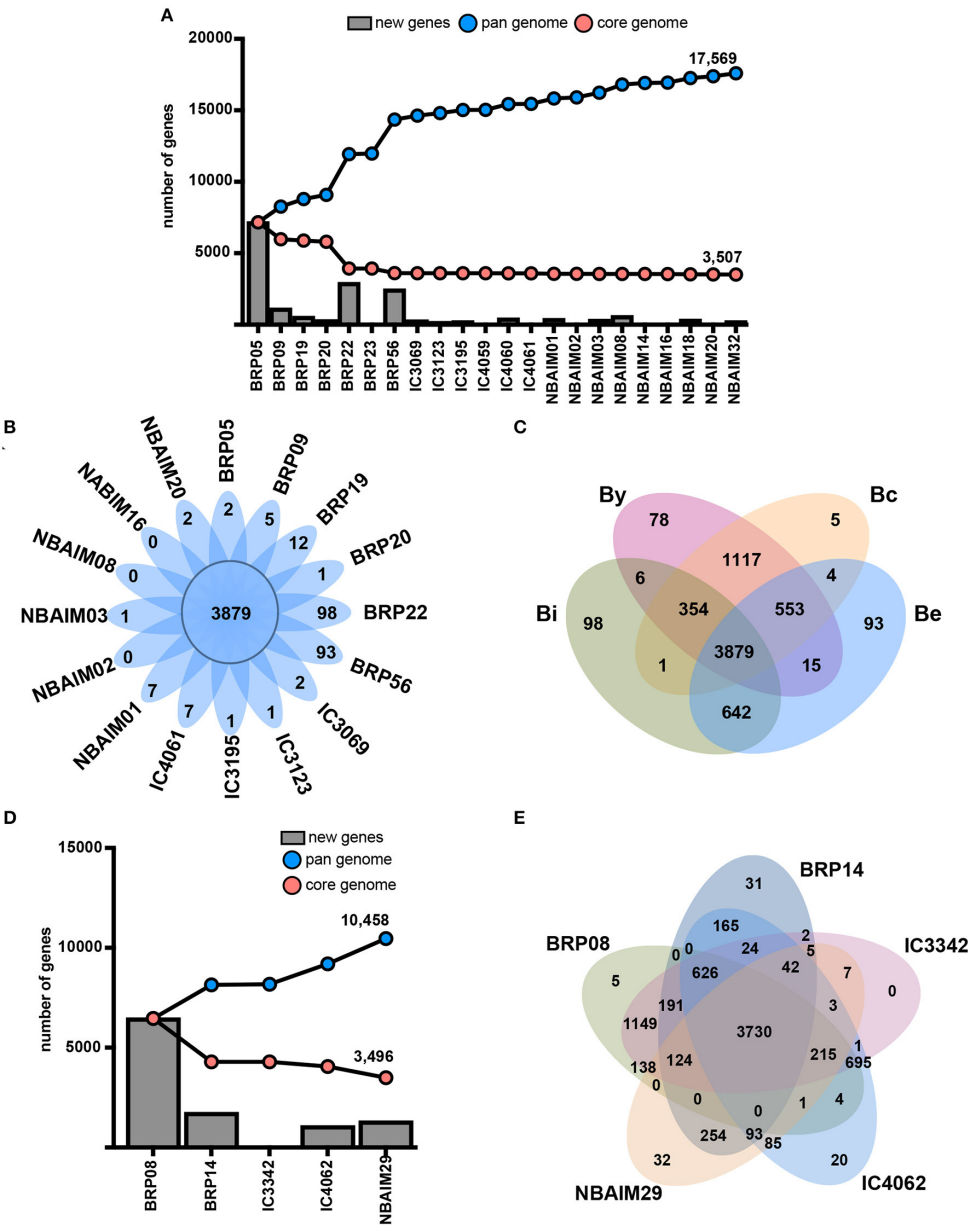
Genetic features. **(A)** Core genome and pangenome of *Bradyrhizobium* where X-axis shows strains and the Y-axis, the number of genes. Blue line: pangenome. Red line: core genome. New genes: bars. **(B)** Core and exclusive orthologue clusters for each *Bradyrhizobium* strain. **(C)** Venn diagram showing shared and exclusive orthologue families among nodulation-type groups. By: the *B. yuanmingensenod*-type group. Bc: the *B. cajaninod*-type group. Be: the *B. elkaniinod*-type group. Bi: the *B. icensenod*-type group. **(D)** Coregenome and pangenome of *Ensifer* where X-axis shows strains and the Y-axis, the number of genes. Blue line: pangenome. Red line: core genome. New genes: bars. **(E)** Core and exclusive orthologue clusters for each *Ensifer* strain.

Putative proteomes of *Bradyrhizobium* and *Ensifer* IU and IC strains were analysed to infer their core genome and pangenomes (Figures 8A,D). Both groups of strains showed an open pangenome of 17,596 and 10,458, respectively, and a core genome of *circa* 3,500 genes for both. Non-core genes present in each group of strains could play a role in soil endurance, competition for root colonisation, host specificity, or symbiosis establishment and, therefore, may help explain the differences observed in plant growth promotion (Figure 3).

#### Bradyrhizobium Orthologues

To reduce the complexity and computational time, we chose representative strains (shown in bold) between those sharing ANIm values greater than 99% similarity: *B. yuanmingense* **BRP05**-BRP23 (99.91%), **IC3069**-IC4069 (99.86%), **NBAIM03**-NBAIM14 (99.18%), NBAIM32-IC4060-**IC4061** (99.27-99.28%), and NBAIM18-**NBAIM20** (99.89%). All IU and IC strains share a total of 3,879 orthologue clusters with enrichment of DNA-related functions (GO:0006412, GO:0006313, and GO:0003700), transmembrane transport (GO0055085 and GO:0008643), and cell shape regulation (GO:0008360) (Figure 8B). Thirteen out of 16 analysed strains show exclusive orthologue clusters, which are not present in any other strain. *Bradyrhizobium* sp. BRP22 and *B. brasilense* BRP56 show the greatest number of exclusive groups of orthologues, 98 and 93, respectively (Figure 8B), which could be the result of the phylogenomic differences with other *B. yuanmingense* strains (Figures 2, 4, 5).

Since the most distinct feature observed among IU and IC strains is the nodulation-related gene groups (Figure 5, Supplementary Figure S3), we have compared the orthologue clusters shared among By, Bc, Be, and Bi (Figure 8C). The exclusive genes for each group represent orthologue clusters that are present in all strains within that specific group. By is the only group that exclusively shows enrichment in GO functions for carbohydrate transport (GO: 0008643 and GO: 0015407). Among the 1,117 orthologue groups shared between By and Bc strains there is enrichment in clusters associated with chemoreceptors (GO: 0007165), permeases (GO: 005585), and flagellum-dependent cell motility (GO: 0071973), which are not present in *Bradyrhizobium* sp. BRP22 and *B. brasilense* BRP56. Nevertheless, these strains could partially compensate for this absence through the catabolism of aromatic compounds (GO: 0019439), which are a component of pigeon pea root exudates (Ae et al., 1990).

#### Ensifer Orthologues

We compared the orthologues groups of IU and IC *Ensifer* strains, which all share 3,730 orthologue clusters (Figure 8E). In this core set, there is enrichment in different DNA-related biological processes (GO: 0006412, GO: 0006313, and GO: 0035556) and transmembrane transport (GO: 0055085). The comparison between IU and IC strains shows enrichment for IC strains in an orthologue cluster annotated as putative adenylate cyclase 3 (*cya3*, GO: 0035556), which could be the reason for the significantly different plant performance between IC and IU strains (Figure 3). *Ensifer* sp. IC3342, *Ensifer* sp. IC4062, *Ensifer* sp. BRP08, and *E. aridi* BRP14 strains shared 626 orthologues families that are not present in *E. alkalisoli* NBAIM29 (Figure 8E). Within this group of orthologues, there is enrichment in the biosynthesis pathway of rhizobactin 1021 (GO: 0019289). Since *E. alkalisoli* NBAIM29 has a significantly better plant performance (Figure 3), we hypothesise that synthesis of rhizobactin 1021 might be a cost, which *E. alkalisoli* NBAIM29 would not sustain.

### Genotype-Metadata Correlation

**Figure 9:**
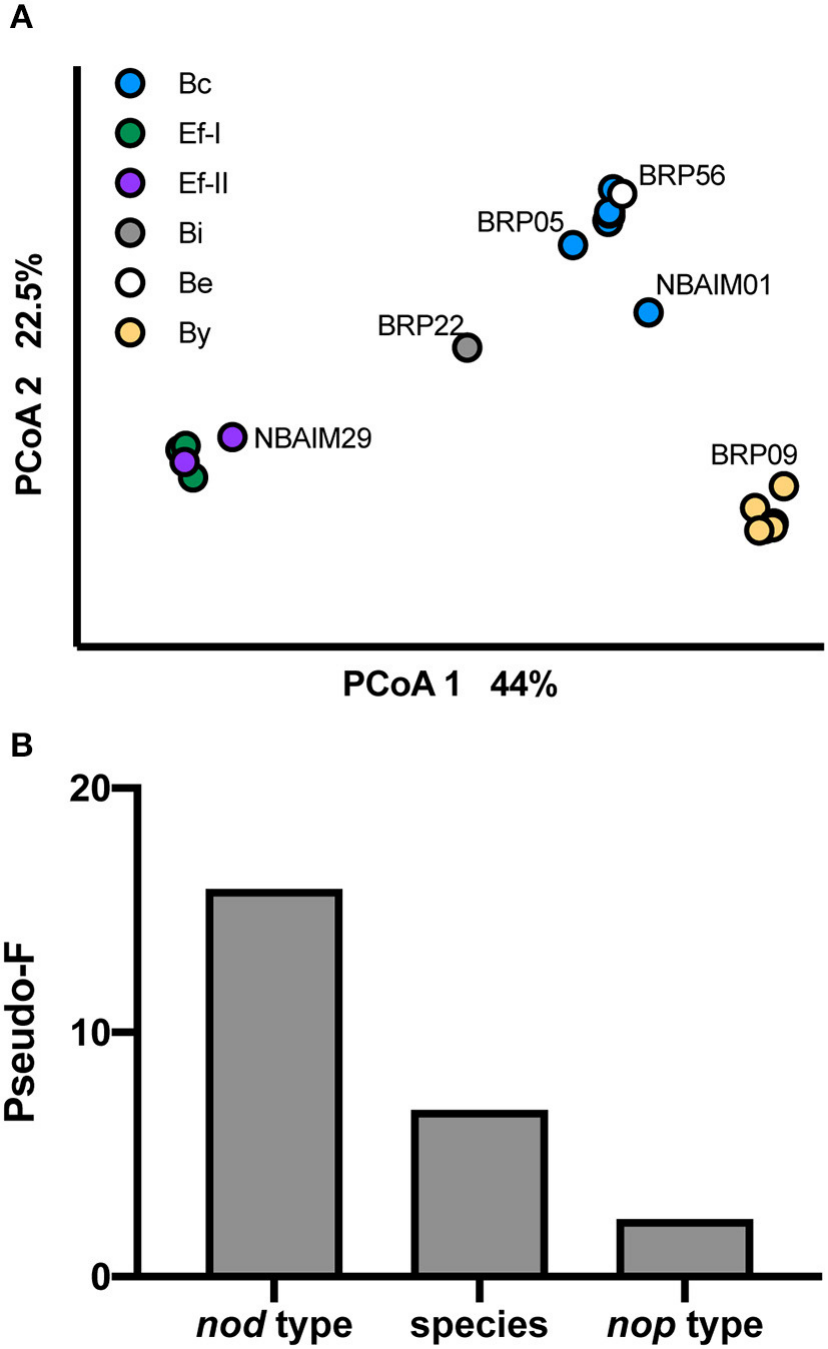
Population structure. **(A)** PCoA representing pigeon pea population and shown with visual separation by *nod* type. **(B)** Influence of different factors on pigeon pea endosymbiont population using PERMANOVA. Pseudo-F value as a proxy. *N* = 27.

We have analysed the pigeon pea population structure based on GC%, genome length, number of tRNAs, rRNA clusters, *repABC, nod* type, and presence/absence of *nod* and *nop* genes (Figure 9). The population separates based on the *nod* group each strain belongs to (Figure 9A). In addition, we have run PERMANOVA using bacterial species (*B. yuanmingense, Bradyrhizobium* sp., *Ensifer* sp., *E. alkalisoli*, and *E. aridi*), *nod* type (By, Bc, Be, Bi, Ef-I, and Ef-II), *nop* profile (B-*nop*-I, B-*nop*-II, B-*nop*-II, B-*nop*-IV, B-*nop*-V, B-*nop*-VI, B-*nop*-VII, E-*nop*-I, E-*nop*-II, E-*nop*-III, and E-*nop*-IV), location of isolation (Madhya Pradesh, Uttar Pradesh, Punjab, Haryana, Tamil Nadu, and Maharashtra), the plant host from which the strain was originally isolated (*C. cajan* cv. Asha, *C. cajan* cv. Bahar, *Indigofera glandulosa, Arachis hypogaea, Macroptilium atropurpureum*, and *Pongamia pinnata*), and the culture collection (or origin) (BRP, IC, and NBAIM) (Figure 9B). The main factor controlling the assembly of the pigeon pea endosymbiont population is the type of *nod* genes, followed by the species the strain belongs, to and finally, the *nop* gene set that each strain contains. The factors that had no significance were the location of isolation, the plant host from which the strain was originally isolated, and the culture collection from which the strain came (origin).

## Discussion

Among the BOX-PCR-reduced population of 65 strains, only 19 were able to establish symbiosis with pigeon pea under laboratory conditions. The isolation of non-symbiotic bacteria from nodule samples has previously been reported (Wu et al., 2011), as well as opportunistic infection (Zgadzaj et al., 2015). Moreover, Fossou et al. (2016) in their sampling of nodule-isolated strains from pigeon pea in Ivory Coast showed that 22% of the population did not display any rhizobia-like features. In addition, they were unable to amplify nitrogenase-encoding sequences (*nifH*) from 5% of the selected strains.

In our pigeon pea endosymbiont population of 27 IU and IC strains, the diversity is moderately driven by the species the strain belongs to (Figure 9). The main species-nodulating pigeon pea in India is *B. yuanmingense* (20 out of 27, Figure 2). In their study of pigeon pea endosymbionts in the Dominican Republic, Araújo et al. (2015) found that all strains investigated had a 99.8% identity to *B. yuanmingense* CCBAU 10071^T^. Moreover, one of the sequenced pigeon pea endosymbionts from this study, *B. yuanmingense* 3051 (ALSPC3051), shows a high ANIm similarity (95.7–96%) to *B. yuanmingense* IU and IC strains (Figure 2, Supplementary Table S7). It is interesting to note that *B. yuanmingense* may be a predominant symbiont in India since it has been isolated from several legumes throughout the country (Ojha et al., 2017; Rathi et al., 2018). *B. brasilense* BRP56 and *Bradyrhizobium* sp. BRP22 are rare strains in this population; however, within this superclade II exist other pigeon pea-nodulating species isolated in the Dominican Republic, Brazil, and Ethiopia, such as *B. ivorense* and *B. elkanii* (Stepkowski et al., 2003; Wolde-Meskel et al., 2005; Fossou et al., 2016, 2020).

Nodulation-related genes are the main diversity driver in the pigeon pea endosymbiont population (Figure 9). The *nodC* sequences defined clear groups, with Bc the most common (12 out of 27). This sequence is similar to the reference strains, including *B. zhanjiangense* CCBAU 51778^T^, a Chinese strain isolated from *A. hypogaea* (Li et al., 2019), and *B. cajani* AMBPC1010^T^, a *C. cajan* strain isolated in the Dominican Republic (Araújo et al., 2017). Despite differences observed in *nodC*, all *Bradyrhizobium* spp. share a common *nod* cluster *nolA*-[]-*nodD2*-[]-*nodD1*-[]-*nodABCSUIJ*-[]-*nodZ* (Supplementary Figure S3). However, different presence/absence patterns were observed for genes related to NF modifications, *nolY, nodK, nodY, nolO, nolN, nolL*, or *noeE* for each *Bradyrhizobium nodC* type group, By, Bc, Be, and Bi (Figures 4, 5). The IU and IC *Bradyrhizobium* strains either have *nodY* or *nodK* between nodD1 and *nodA* (Supplementary Figure S3), whose functions have not yet been elucidated (Menna and Hungria, 2011). The IU and IC strains from the Bc and Be groups have a *nolY* homologue upstream from *nolA* (Figures 5B,C). In *B. diazoefficiens* USDA 110^T^, a *nolY* mutant showed a significant disadvantage in nodule kinetics on *Vigna radiata* (mung bean), but this detrimental effect was not so strong in soybean (Dockendorff et al., 1994), suggesting that the presence or absence of *nolY* in *Bradyrhizobium* spp. could be related to their host range. *Bradyrhizobium* sp. BRP22 is the only strain with *nolL* and *noeE* homologues, which seem to modify the NF playing a role in host specificity (Figure 5D) (Corvera et al., 1999; Wei et al., 2008). Moreover, differences were observed in the presence and absence of the annotated carbamoyltransferases *nolN* and *nolO* (Supplementary Figure S3). Bc, Be, and Bi strains have the *nolO* homologue between *nodJ* and *nodZ*, whereas By strains have *nolN*. In *B. diazoefficiens* USDA 110^T^ *nolMNO* is part of the *nod* operon, and *nolNO*, together with *nodZ*, acts in the NF 2-O-methylfucosylation. However, mutants in *nolO* or *nolNO* in this strain showed the same phenotype: delayed nodule formation and a reduced percentage of nodules per plant in legumes like soybean or mung bean (Luka et al., 1993). Since both genes encode carbamoyltransferases, which probably undertake the same function, it is possible that NF 2-O-methylfucosilation is essential to establish symbiosis in pigeon pea. Since differences in plant performance were not observed (Supplementary Figure S4B), we conclude that the common functional *nod* cluster for IU and IC *Bradyrhizobium* spp. is *nolA*-*nodD2D1-nodY/K*-nodABCSUIJ-*nolO/nolN*. Genes like *nodY, nodK, noeE*, and *nolL* only reflect phylogenetic diversity among these strains. The absence of *nolY* could have been positively selected, since the major group, Bc, does not show a *nolY* homologue. However, this selection has no impact on plant performance (Supplementary Figure S4A).

Based on the orthologue analysis, each *Bradyrhizobium* group could have developed a different strategy to endure in the pigeon pea rhizosphere (Figure 8C). Bc is the most common pigeon pea symbiont group in our population, and we hypothesise that it may be better adapted to the pigeon pea root environment. Together with By, both have homologues of chemoreceptors and flagella, which Be and Bi do not possess. These groups of genes are essential in rhizobium-legume symbioses (Jiang et al., 2016; Wheatley et al., 2020). However, *Bradyrhizobium* sp. BRP22 and *B. brasilense* BRP56 could partially compensate for this absence through the catabolism of aromatic compounds, which are present in pigeon pea root exudates (Ae et al., 1990). The By group shows enrichment in carbohydrate transporters. Since sugars are one of the main components of root exudates (Lynch and Whipps, 1990), it is possible that having a greater pool of carbohydrate transporters could give the By group an adaptative advantage in the pigeon pea rhizosphere, resulting in their increased prevalence in nodules.

Among IU and IC *Bradyrhizobium* spp., the Bc strain *B. yuanmingense* BRP05 promotes plant growth more significantly than any other strain tested (Figure 3), which could be, in part, a result of the presence of T3SS pili structures like *nopA* (Figure 7A). NopA is part of the external T3SS apparatus, and its deletion completely abolishes the secretion of other Nops, since it is a major component of the T3SS pili (Krishnan et al., 2003). However, in *E. fredii* USDA 257, the absence of *nopA* extends the host range to other soybean varieties, whereas, in cowpea, it has a slightly deleterious effect (Kim and Krishnan, 2014). Nevertheless, it is impossible to draw firmer conclusions without further strains showing similar characteristics to *B. yuanmingense* BRP05.

*Ensifer* spp. is an infrequent pigeon pea endosymbiont in the population since only five (of 22) IU and IC strains were assigned to this genus. There are a few records of *Ensifer* strains,-nodulating pigeon pea, including strains isolated in Cerrado soil in Brazil and India (Coutinho et al., 1999; Stepkowski et al., 2003). Their rarity is probably related to pigeon pea specificity rather than low *Ensifer* spp. numbers in soil, since, in India, *Ensifer* spp. are common endosymbionts of native legumes growing in alkaline soils (Gehlot et al., 2013; Tak et al., 2016; Sankhla et al., 2017; Rathi et al., 2018; Choudhary et al., 2020). Regarding nodulation genes, the most relevant feature is *nolL*, where its absence correlates with a significantly improved plant performance in *E. alkalisoli* NBAIM29 (Figures 3, 6). *nolL* determines 4-O-acetylation of the fucosyl residue in NF, and its deletion has been shown to have a negative effect on *R. etli* CE3 nodule kinetics in some *Phaseolus vulgaris* cultivars and in *V. umbellata* (Corvera et al., 1999). Furthermore, the heterologous expression of *nolL* in *E. fredii* USDA 257 extends its host range to other legumes like *Leucaena leucocephala* and *L. halophilus* (Berck et al., 1999). NolL plays a role in both host specificity and host range. Therefore, we hypothesise that the lack of the NF fucosyl acetylation might give *E. alkalisoli* NBAIM29 an advantage in plant recognition, explaining the phenotype observed *in planta* (Figure 3). However, with only one strain, this is highly speculative. Moreover, *E. alkalisoli* NBAIM29 is the only IU and IC *Ensifer* spp. strain that has *nopC, nopP*, and *nopT*; all of them are well-characterised T3SS effectors with functions related to host-range and interaction with the plant immune system. The deletion of any of these *nop* genes results in a reduction of nodules in the symbiosis between *E. fredii* and different legumes (soybean and *P. vulgaris*) (Boundy-Mills et al., 1994; Skorpil et al., 2005; Dai et al., 2008; López-Baena et al., 2009).

The comparison of orthologue groups between the IU and IC *Ensifer* spp. strains showed an exclusive group in IC annotated as an adenylate cyclase 3 (cya3), which modulate the extent of epidermal infection during nodulation (Tian et al., 2012). Indeed, a mutation in *cya3* (*cya5*) in *E. meliloti* CXM1-105 significantly increased alfalfa shoot dry weight (Sharypova et al., 1999), which could be reflected in the different plant performances between IC and IU strains (Figure 3).

Remarkably, *E. alkalisoli* NBAIM29 lacks an orthologue family related to the synthesis of rhizobactin 1021 (*rhbBCDEF*), a siderophore that chelates iron (Fe) (Lynch et al., 2001). We hypothesise that the biosynthesis of a siderophore might be redundant in the pigeon pea rhizosphere since it exudes piscidic acid, an aromatic compound that solubilises phosphorous (P) by chelating Fe from P-Fe compounds (Ae et al., 1990). Siderophore biosynthesis would represent a metabolic cost to the other *Ensifer* strains, *Ensifer* sp. IC3342, *Ensifer* sp. IC4062, *Ensifer* sp. BRP08, and *E. aridi* BRP14, and could explain their significantly lower plant performance compared to that of *E. alkalisoli* NBAIM29 (Figure 3). It is possible that the presence of these Nop proteins, together with the lack of *nolL* and the rhizobactin 1021 biosynthesis pathways, endows *E. alkalisoli* NBAIM29 with improved plant recognition machinery that could translate into better performance with pigeon pea, but without further strains showing the same characteristics, it is impossible to tell at this stage.

Due to its intrinsic capacity to tolerate drought (grown on drylands), pigeon pea is a promising candidate for resilience to climate change; however, its yield remains low. The use of symbionts well-adapted to the growth conditions of pigeon pea could increase its productivity (Pellegrino et al., 2011; Pellegrino and Bedini, 2014; Pastor-Bueis et al., 2019). Our findings demonstrate that the most common pigeon pea endosymbiont in India is a *B. yuanmingense* strain with a *B. cajani-B. zhanjiangens* (Bc) *nod* type, defined mainly by the absence of *nolY* and the presence of *nolO*. Since we have not observed location to be a driving factor in population diversity, our findings may apply to much, if not all, of India. Due to its intrinsic capabilities for persisting and establishing symbiosis, in addition to its genetic and genomic features, we suggest that *B. yuanmingense* BRP05 could be a good candidate for inclusion in inoculum formulations for pigeon pea in India. However, testing a range of IU strains for symbiotic performance in field trials is essential to assess their real-world performance. Moreover, the less common *Ensifer* strains, like *E. alkalisoli* NBAIM29, may be better for alkaline conditions, where members of this genus often perform well (Gehlot et al., 2013; Tak et al., 2016; Sankhla et al., 2017; Rathi et al., 2018; Choudhary et al., 2020).

This study presents a first step in defining and collecting strains that can nodulate pigeon pea in Indian soils. Their ability to influence plant performance has been investigated in glasshouse experiments under sterile conditions. Therefore, extensive trialling in the field in India, using a range of different varieties of pigeon pea, is now suggested to evaluate their performance under these agronomic conditions. We are confident that such studies will lead to the selection of a group of highly effective strains for use in inoculant technology, improving the symbiotic performance of this essential legume in India.

## Data Availability Statement

The data presented in the study are deposited in GenBank repository, BioProject PRJNA679722.

## Author Contributions

BJ, PP, DR, AS, AP, and VR conceived the study and designed the manuscript. BJ, MM, AB, DC, SP, NA, SM, SK, PS, and MK performed the experiments. BJ, MM, and AT analysed the data. BJ prepared the manuscript. BJ, AT, AE, VR, EJ, PP, MM, DR, AP, AS, and AB critically reviewed the manuscript. All authors listed have made a substantial, direct and intellectual contribution to the study, and approved it for publication.

## Funding

This study was supported by the Biotechnology and Biological Sciences Research Council [Grant No. BB/N013387/1], granted to PP; and by the Department of Biotechnology, Government of India [Grant No. BT/IN/UK-VNC/41/DLN/2015-16], granted to DR, AS, and AP.

## Conflict of Interest

The authors declare that the research was conducted in the absence of any commercial or financial relationships that could be construed as a potential conflict of interest.

## Publisher's Note

All claims expressed in this article are solely those of the authors and do not necessarily represent those of their affiliated organizations, or those of the publisher, the editors and the reviewers. Any product that may be evaluated in this article, or claim that may be made by its manufacturer, is not guaranteed or endorsed by the publisher.
