# Supplementary material for: Genomic Diversity of Pigeon Pea (Cajanus cajan L. Millsp.) Endosymbionts in India and Selection of Potential Strains for Use as Agricultural Inoculants
Source: Front Plant Sci. 2021 Sep 7;12:680981. doi: 10.3389/fpls.2021.680981 (PMC8453007; doi:10.3389/fpls.2021.680981)
Supplement: Supplementary file 2 [file Image_2.pdf]

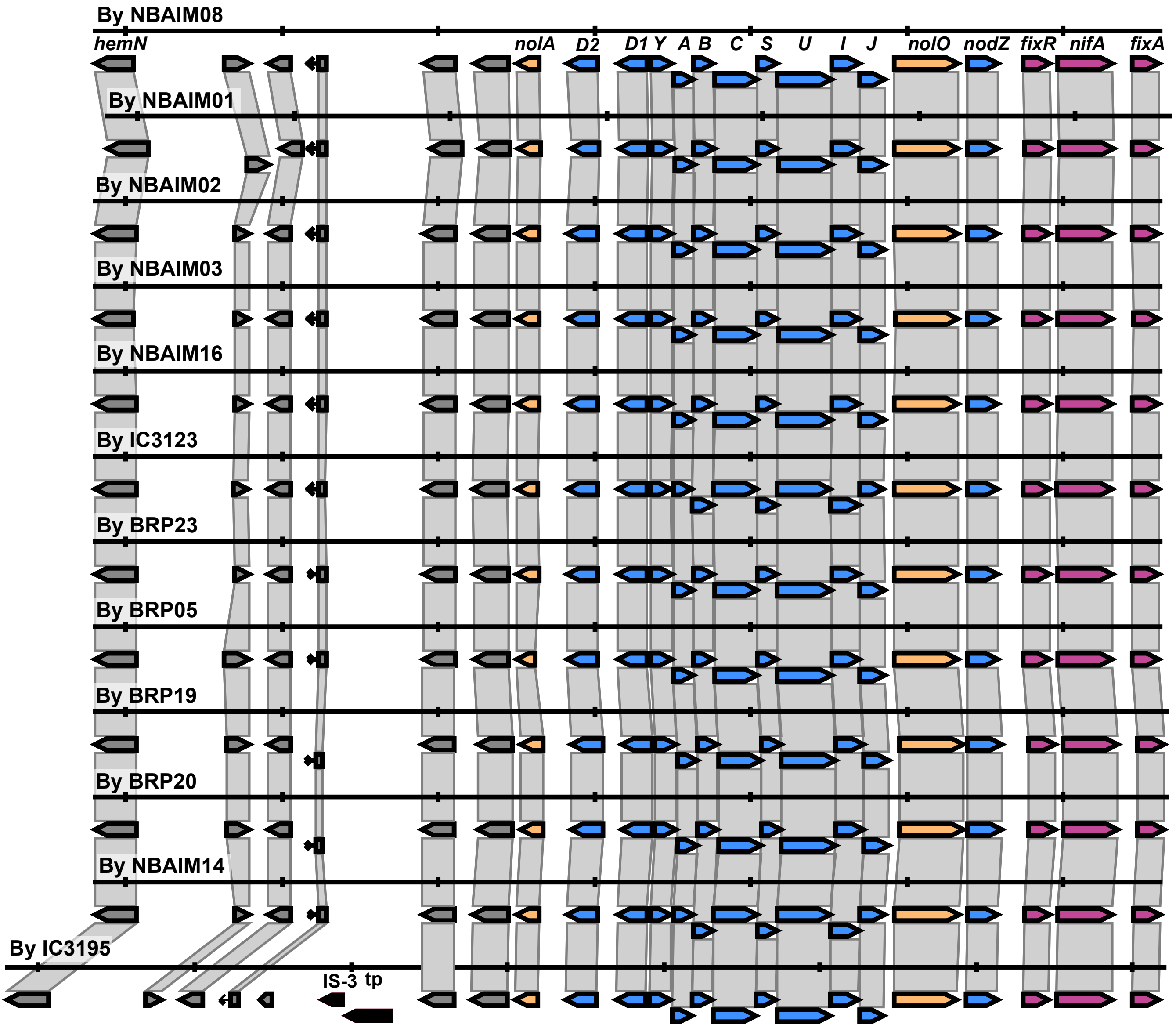

Supplementary Figure S2. The *nod* cluster synteny of IU and IC strains in the Bc group. Each row represents a single strain and shows the *nod* cluster organisation and its genomic context. The colour of the arrow reflects the genes: blue for *nod*, yellow for *nol*, pink for *fix-nif* and black for transposases/insertion-related genes. Vertical markers indicate 5Kb in each genome.
