# Supplementary material for: Genomic Diversity of Pigeon Pea (Cajanus cajan L. Millsp.) Endosymbionts in India and Selection of Potential Strains for Use as Agricultural Inoculants
Source: Front Plant Sci. 2021 Sep 7;12:680981. doi: 10.3389/fpls.2021.680981 (PMC8453007; doi:10.3389/fpls.2021.680981)
Supplement: Supplementary file 3 [file Image_3.pdf]

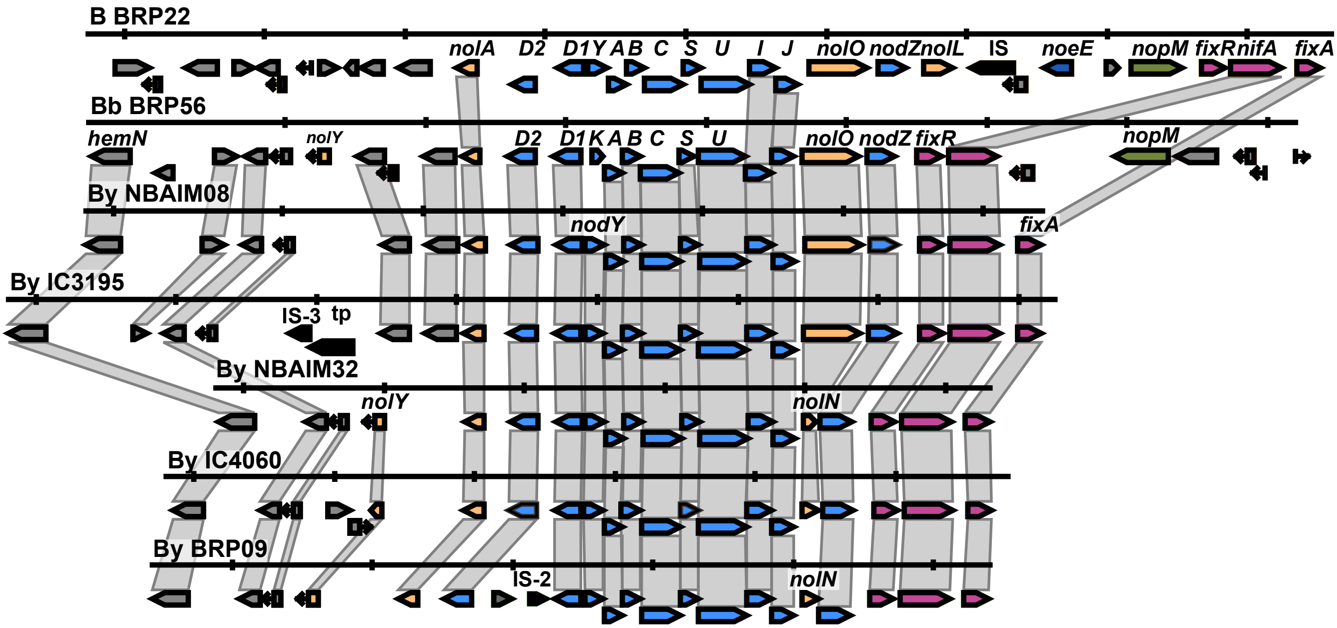

Supplementary Figure S3. The nod cluster synteny of representative IU and IC strains in Bradyrhizobium. Each row represents a single strain and shows the nod cluster organisation and its genomic context. The colour of the arrow reflects the genes: blue for nod, yellow for nol, pink for fix-nif and black for transposase/insertion-related genes. Vertical markers indicate 5Kb in each genome.
