# Supplementary material for: Genomic Diversity of Pigeon Pea (Cajanus cajan L. Millsp.) Endosymbionts in India and Selection of Potential Strains for Use as Agricultural Inoculants
Source: Front Plant Sci. 2021 Sep 7;12:680981. doi: 10.3389/fpls.2021.680981 (PMC8453007; doi:10.3389/fpls.2021.680981)
Supplement: Supplementary file 4 [file Image_4.pdf]

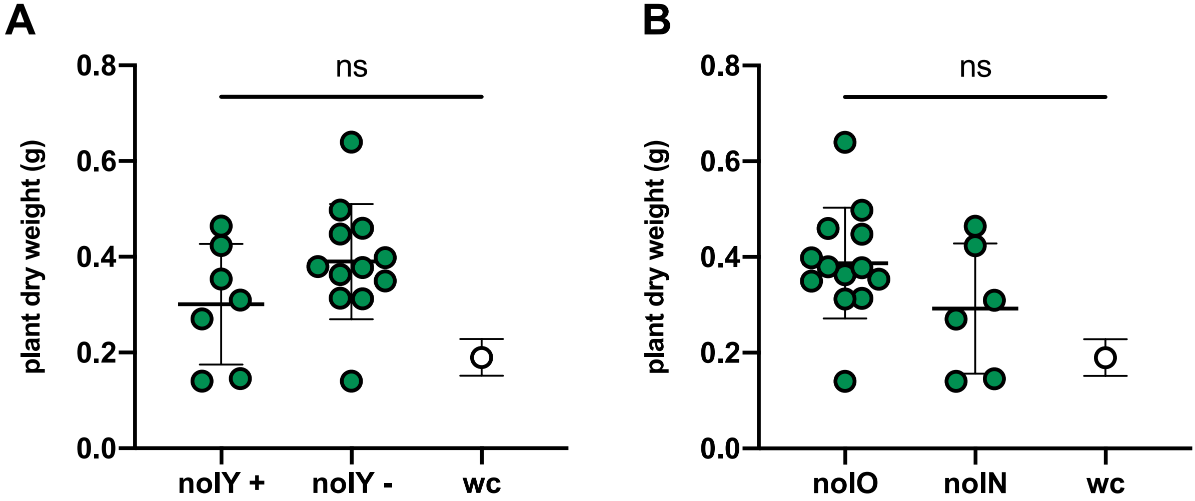

Supplementary Figure S4. A comparison of the *nod* cluster synteny within the Bradyrhizobium spp. and effect on plant growth. A) plant dry weight plotted againts presence and absence of *nolY*. B) plant dry weight plotted against presence of *nolO* or *nolN*. Each dot represents the average of 5 biological replicates. Error bars represent standard error of the mean. Significant differences were assessed across samples and indicated by horizontal lines. ns: no significant. Analysis was performed using ANOVA with Sidak's post-hoc test. wc: watre control.
